# Increased risk of Alzheimer’s disease among patients with age-related macular degeneration: A nationwide population-based study

**DOI:** 10.1371/journal.pone.0250440

**Published:** 2021-05-07

**Authors:** Li-Yen Wen, Lei Wan, Jung-Nien Lai, Chih Sheng Chen, Jamie Jiin-Yi Chen, Ming-Yen Wu, Kai-Chieh Hu, Lu-Ting Chiu, Peng-Tai Tien, Hui-Ju Lin

**Affiliations:** 1 School of Chinese Medicine, China Medical University, Taichung, Taiwan; 2 Department of Medical Laboratory Science and Biotechnology, Asia University, Taichung, Taiwan; 3 Department of Obstetrics and Gynecology, China Medical University Hospital, Taichung, Taiwan; 4 Division of Chinese Medicine, Asia University Hospital, Taichung, Taiwan; 5 Department of Ophthalmology and Department of Molecular Genetics, China Medical University Hospital, Taichung, Taiwan; 6 Management office for Health Data, China Medical University Hospital, Taichung, Taiwan; 7 Graduate Institute of Clinical Medical Science, College of Medicine, China Medical University, Taichung, Taiwan; National Yang-Ming University Hospital, TAIWAN

## Abstract

**Objective:**

This study aimed to investigate the risk of Alzheimer’s disease among patients with age-related macular degeneration and its association with confounding comorbidities.

**Method:**

This was a population-based, retrospective cohort study. By accessing data from the National Health Insurance Research Database of Taiwan, we identified 10,578 patients aged 50–100 years who were newly diagnosed with age-related macular degeneration between 2000 and 2012 and 10,578 non- age-related macular degeneration individuals. The comorbidities assessed were osteoporosis, diabetes, cirrhosis, cerebrovascular disease, chronic kidney disease, hypertension, hyperlipidemia, coronary artery disease, and chronic obstructive pulmonary disease.

**Results:**

Patients with age-related macular degeneration had a 1.23-fold increased risk of their condition advancing to Alzheimer’s disease (aHR = 1.23, 95% CI = 1.04–1.46). The younger patients were diagnosed with age-related macular degeneration, the more likely patients got Alzheimer’s disease (50–64 age group: aHR = 1.97, 95% CI = 1.04–3.73; 65–79 age group: aHR = 1.27, 95% CI = 1.02–1.58; 80–100 age group: aHR = 1.06, 95% CI = 0.78–1.45). In addition, there were significantly higher risks of Alzheimer’s disease for patients with cirrhosis (aHR = 1.50, 95% CI = 1.09–2.06) in the age-related macular degeneration cohort than in the non-age-related macular degeneration cohort.

**Conclusion:**

Patients with age-related macular degeneration may exhibit a higher risk of Alzheimer’s disease than people without age-related macular degeneration.

## Introduction

Alzheimer’s disease (AD) is a neurodegenerative disorder characterized by progressive and irreversible cognitive impairment. AD is the most common cause of dementia in the elderly, with an estimated 44 million people having AD worldwide, and the prevalence is predicted to triple by 2050 [1]. The characteristic neuropathological change in AD is the presence of amyloid-beta (Aβ) plaques and neurofibrillary tangles in brain tissues [2, 3].

The exact etiology and pathogenesis of AD remain unclear. Over the past two decades, the amyloid cascade hypothesis has been one of the most acceptable theories [4, 5]. Aβ stems from a transmembrane protein of neuronal cells termed amyloid precursor protein (APP). Aβ is generated when APP is sequentially cleaved by β-secretase and γ-secretase [6, 7]. The production and clearance imbalance of Aβ results in abnormal Aβ deposition in the neuropil, which is considered the main cause of synaptic loss and neuronal cell death in AD [8, 9].

Age-related macular degeneration (AMD) is another neurodegenerative disease that mainly affects the aging population. The disease results in progressive central vision loss and is the fourth leading cause of blindness worldwide [10, 11]. The hallmark pathology of AMD is ocular drusen and extracellular deposits located between the retinal pigment epithelium (RPE) and Bruch’s membrane [12]. Similar to senile plaques in AD, Aβ is also an important constituent in drusen [13–15]. Previous study has demonstrated that the Aβ in drusen correlates with the location of photoreceptors and RPE cell degeneration [16]. Moreover, oxidative stress, neuroinflammation, and complement activation have been implicated in both AD and AMD [17–19]. AD and AMD share risk factors such as cigarette smoking and diabetes [20–23]. In addition, genetic studies have also noted that the apolipoprotein E (APOE) epsilon‑4 and cysteine proteinase inhibitor cystatin C gene (CST3) increase the risk for both disorders [24, 25].

Because of the similarity in pathologic features between AMD and AD, there has been an increasing interest in investigating the potential link between the two diseases in recent years. However, most studies on the association between AMD and AD have shown paradoxical results. Several publications have documented that AMD was associated with cognitive impairment and dementia [26–29]. On the contrary, an epidemiologic study reported that there was no significant increase of the risk of dementia and AD following AMD [30]. Most past studies contained small sample size or only adjusted for a few factors. Therefore, we used a national population-based dataset from Taiwan National Health Insurance to investigate the association between AMD and AD.

## Methods

### Data resource

The requirement for informed consent was waived by both the National Health Insurance (NHI) Administration and China Medical University Hospital Research Ethics Committee.

Taiwan adopted a single-payer NHI program in March 1995. It is mandatory for Taiwanese citizens to enroll in the program; the coverage rate of NHI is over 99 percent. We used the Longitudinal Health Insurance Database 2010 (LHID 2010), which contains the claims data of 1,000,000 beneficiaries randomly sampled from the National Health Insurance Research Database (NHIRD). No significant differences in age and sex were found between the LHID 2010 and the enrollees in the mother NHIRD. Patient demographic characteristics included encrypted identification numbers, sex, dates of birth and death, diagnostic data, and procedures. The diagnostic data were coded according to the International Classification of Diseases, Ninth Revision, Clinical Modification (ICD-9-CM). The accuracy of the diagnosis codes of common conditions in NHIRD has been validated [31–33]. This study was approved by China Medical University Hospital Research Ethics Committee.

### Sample participant, outcome, and comorbidity

We included 10,578 individuals aged at 50–100 years who were newly diagnosed with AMD (ICD-9-CM code 362.50, 362.51, and 362.52) by certified ophthalmologists between 2000 and 2010 in the AMD group. The date of the first outpatient visits or inpatient visit for AMD was defined as index date. For comparison, each AMD patient was frequency matched with an individual without AMD based on gender, age (in 5-year bands), and index-year. The exclusion criteria was patients with a pre-existing diagnosis of AD (ICD-9-CM 331) in both AMD cohort and control cohort. All study subjects were followed up until the appearance of AD, death, withdrawal from the insurance program or the end of study period (December 31, 2013).

All included patients were followed from the index date until the occurrence of the endpoint, withdrawal from the NHI program, or the end of 2013. The potential comorbidities included osteoporosis (ICD-9-CM code 733), diabetes (ICD-9-CM code 250 and A181), cirrhosis (ICD-9-CM code 571 and A347), cerebrovascular disease (ICD-9-CM code 342.34, and 430–438), chronic kidney disease (ICD-9-CM code 403.11, 403.91, 404.12, 404.92, 585, 586, v42.0, v45.1, v56.0, and v56.8), hypertension (ICD-9-CM code 401–405, A260 and A269), hyperlipidemia (ICD-9-CM code 272), coronary artery disease (ICD-9-CM code 410–414), and chronic obstructive pulmonary disease (ICD-9-CM code 491, 492, 493, and 496).

### Statistical analysis

Descriptive statistics of demographics and comorbidities were summarized by counts, percentages, averages, and standard deviations (SDs) for the AMD and non-case cohort. Chi-square and Wilcoxon rank-sum tests were used to examine the differences in the characteristic distributions between the two cohorts. The cumulative incidence for AD was estimated using Kaplan-Meier methods and significance of the difference between the AMD cohort and the non-case cohort were tested using log-rank test. The incidence rate was calculated as the number of events divided by the person-years during the follow-up. The univariate Cox proportional hazards model was used to compute the hazard ratio (HR) and the 95% confidence interval (CI) for AD in the AMD cohort when compared to the non-case cohort. And the multivariate Cox proportional hazards model with covariates of gender, age, and comorbidities was used to compute the adjusted hazard ratio (aHR) and the 95% CI for AD in the AMD cohort when compared to the non-case cohort. Data analysis in this study was performed using SAS statistical software (Version 9.4 for Windows; SAS Institute Inc., Cary, NC, USA), with statistical significance set at a p value < 0.05.

## Results

Table 1 reveals the demographic characteristics and the prevalence of comorbidities in both cohorts. The mean ages in the AMD and non-AMD group were 70.5 (±9.54, standard deviation [SD]) and 70.4 (±9.60, SD) years old, respectively, with the mean follow-up time of 5.68 (±3.81, SD) and 5.48 (±3.84, SD) years. The presence of osteoporosis, diabetes, cirrhosis, cerebrovascular disease, chronic kidney disease, hypertension, hyperlipidemia, coronary artery disease and chronic obstructive pulmonary disease was significantly higher in patients with AMD than controls.

**Table 1:** Demographic characteristics and presence of comorbidities in patients with and without age-related macular degeneration (AMD).

	AMD (n = 10578)		Non-AMD (n = 10578)		*p*-Value*
	n	%	N	%	
**Gender**					>0.99
Male	5208	49.2	5208	49.2	
Female	5370	50.8	5370	50.8	
**Age, years**					>0.99
50–64	3106	29.4	3106	29.4	
65–79	5661	53.5	5661	53.5	
80–100	1811	17.1	1811	17.1	
mean(SD)^†^	70.5 (9.54)		70.4 (9.60)		0.3120
**Comorbidity**					
Osteoporosis	2648	25.0	2092	19.8	<0.0001
Diabetes	5028	47.5	3965	37.5	<0.0001
Cirrhosis	4137	39.1	3220	30.4	<0.0001
Cerebrovascular disease	2832	26.7	2363	22.3	<0.0001
Chronic kidney disease	736	6.96	560	5.29	<0.0001
Hypertension	7557	71.4	6693	63.3	<0.0001
Hyperlipidemia	4738	44.8	3685	34.8	<0.0001
Coronary artery disease	813	7.69	681	6.44	0.0004
Chronic obstructive pulmonary disease	4326	40.9	3596	34.0	<0.0001
**Follow-up time, year**^†^	5.68 (3.81)		5.48 (3.84)		<0.0001

SD, standard deviation.

Diabetes mellitus included type 1 and type 2 diabetes mellitus.

*P-value using *chi-square* for the comparisons between with and without AMD.

^†^Average age and follow-up time using *Wilcoxon rank-sum test* for verification.

Table 2 demonstrates that there was a higher risk of developing AD in the AMD cohort than the control cohort (aHR = 1.23, 95% CI = 1.04–1.46), with incidences of 5.36 and 3.92 per 1,000 person-years in the AMD and non-AMD groups, respectively. Compared to the 50-64-year subgroup, the subgroups with individuals 65–79 years old and more than 80 years old have a significant 3.67 (95% CI = 2.69–5.01) and 7.53 (95% CI = 5.38–10.5) risk for AD. Among all of comorbidities, patients with cerebrovascular disease and chronic obstructive pulmonary disease tended to have a higher risk for the development of AD (aHR = 1.44, 95% CI = 1.19–1.73 and aHR = 1.20, 95% CI = 1.01–1.44). Compared to the patients without any comorbidities, patients with at least one comorbidity also have a significant 1.23 (95% CI = 1.04–1.47) risk for AD.

**Table 2:** Cox proportional HRs for risk of Alzheimer’s disease in patients with and without AMD, in different gender, age groups, and in patients with preexisting comorbidities.

Variable	Alzheimer’s disease			Crude HR (95%CI)	Adjusted HR (95%CI)
	Event	PY	IR		
**AMD**					
No	227	57939	3.92	**1(reference)**	**1(reference)**
Yes	322	60128	5.36	1.36 (1.15–1.62)***	1.23 (1.04–1.46)*
**Gender**					
Female	249	59164	4.21	**1(reference)**	**1(reference)**
Male	300	58903	5.09	1.21 (1.02–1.43)*	0.89 (0.74–1.07)
**Age, years**					
50–64	47	38049	1.23	**1(reference)**	**1(reference)**
65–79	336	65212	5.15	4.22 (3.11–5.72)***	3.67 (2.69–5.01)***
80–100	166	14806	11.2	9.53 (6.88–13.2)***	7.53 (5.38–10.5)***
P for trend				<0.0001	<0.0001
**Comorbidity**					
Osteoporosis					
No	398	94104	4.23	**1(reference)**	**1(reference)**
Yes	151	23963	6.3	1.50 (1.24–1.81)***	1.22 (1.00–1.50)
Diabetes					
No	306	72098	4.24	**1(reference)**	**1(reference)**
Yes	243	45969	5.29	1.25 (1.06–1.48)**	1.06 (0.89–1.26)
Cirrhosis					
No	368	79442	4.63	**1(reference)**	**1(reference)**
Yes	181	38625	4.69	1.01 (0.85–1.21)	--
Cerebrovascular disease					
No	359	93798	3.83	**1(reference)**	**1(reference)**
Yes	190	24269	7.83	2.08 (1.74–2.49)***	1.44 (1.19–1.73)***
Chronic kidney disease					
No	518	112987	4.58	**1(reference)**	**1(reference)**
Yes	31	5080	6.1	1.35 (0.94–1.94)	--
Hypertension					
No	132	42263	3.12	**1(reference)**	**1(reference)**
Yes	417	75804	5.5	1.77 (1.46–2.16)***	1.09 (0.88–1.34)
Hyperlipidemia					
No	365	77597	4.7	**1(reference)**	**1(reference)**
Yes	184	40470	4.55	0.97 (0.81–1.16)	--
Coronary artery disease					
No	511	110964	4.61	**1(reference)**	**1(reference)**
Yes	38	7103	5.35	1.17 (0.84–1.62)	--
Chronic obstructive pulmonary disease					
No	295	78499	3.76	**1(reference)**	**1(reference)**
Yes	254	39568	6.42	1.73 (1.46–2.04)***	1.20 (1.01–1.44)*
**Comorbidity amount**					
0	38	18111	2.09	**1(reference)**	**1(reference)**
≥1	511	99956	5.11	2.46 (1.77–3.43)	1.23 (1.04–1.47)*

PY, person-years; IR, incidence rate, per 1,000 person-years; HR, hazard ratio; CI, confidence interval.

HR adjusted for gender, age, osteoporosis, diabetes, cirrhosis, cerebrovascular disease, chronic kidney disease, hypertension, hyperlipidemia, coronary artery disease, and chronic obstructive pulmonary disease.

*p<0.05

**p<0.01

***p<0.001.

Table 3 presents the risk for AD between patients with and without AMD stratified by gender, age, and comorbidities. Compared with the women without AMD, the AMD women had a significant higher risk of AD (aHR = 1.31, 95% CI = 1.04–1.66). And the younger patients were diagnosed with AMD, the more likely patients got AD (50–64 age group: aHR = 1.97, 95% CI = 1.04–3.73; 65–79 age group: aHR = 1.27, 95% CI = 1.02–1.58; 80–100 age group: aHR = 1.06, 95% CI = 0.78–1.45). Moreover, AMD patients with cirrhosis exhibited 1.50 (95% CI = 1.09–2.06) higher risk of AD compared with the non-AMD group. Comparing to without AMD cohort, the risk of AD for AMD in patients no matter with or without any comorbidities (aHR = 1.19, 95% CI = 1.00–1.42 and aHR = 2.19, 95% CI = 1.13–4.09) were also significantly higher.

**Table 3:** Incidence rates, hazard ratios, and confidence intervals of Alzheimer’s disease in patients with AMD stratified by gender, age and comorbidity.

Variable	AMD						Crude HR (95%CI)	Adjusted HR (95%CI)
	No			Yes				
	Event	PY	IR	Event	PY	IR		
**Gender**								
male	107	29026	3.69	142	30138	4.71	1.27 (0.99-1.64)	1.14 (0.88-1.47)
Female	120	28913	4.15	180	29990	6	1.44 (1.14-1.82)**	1.31 (1.04-1.66)*
**Age, years**								
50-64	14	19125	0.73	33	18924	1.74	2.39 (1.28-4.47)**	1.97 (1.04-3.73)*
65-79	138	31826	4.33	198	33387	5.93	1.36 (1.09-1.69)**	1.27 (1.02-1.58)*
80-100	75	6988	10.7	91	7817	11.6	1.08 (0.79-1.46)	1.06 (0.78-1.45)
**Comorbidity**								
Osteoporosis								
No	168	47959	3.5	230	46144	4.98	1.42 (1.16-1.73)***	1.30 (1.06-1.59)**
Yes	59	9980	5.91	92	13984	6.58	1.11 (0.80-1.55)	1.06 (0.76-1.47)
Diabetes								
No	131	38743	3.38	175	33355	5.25	1.55 (1.23-1.94)***	1.33 (1.06-1.68)*
Yes	96	19196	5	147	26773	5.49	1.09 (0.84-1.41)	1.09 (0.84-1.41)
Cirrhosis								
No	171	41807	4.09	197	37636	5.23	1.28 (1.04-1.57)*	1.13 (0.92-1.40)
Yes	56	16132	3.47	125	22492	5.56	1.60 (1.16-2.19)**	1.50 (1.09-2.06)*
Cerebrovascular disease								
No	150	47548	3.15	209	46250	4.52	1.42 (1.15-1.76)***	1.29 (1.04-1.60)*
Yes	77	10391	7.41	113	13878	8.14	1.10 (0.82-1.48)	1.11 (0.83-1.48)
Chronic kidney disease								
No	214	55876	3.83	304	57111	5.32	1.38 (1.16-1.65)***	1.24 (1.03-1.48)*
Yes	13	2063	6.3	18	3017	5.97	0.94 (0.46-1.93)	1.10 (0.53-2.26)
Hypertension								
No	60	23660	2.54	72	18603	3.87	1.52 (1.07-2.14)*	1.44 (1.02-2.04)*
Yes	167	34279	4.87	250	41525	6.02	1.23 (1.01-1.50)*	1.17 (0.96-1.43)
Hyperlipidemia								
No	164	41081	3.99	201	36515	5.5	1.37 (1.12-1.69)**	1.22 (0.99-1.50)
Yes	63	16858	3.74	121	23613	5.12	1.36 (1.00-1.85)*	1.29 (0.95-1.75)
Coronary artery disease								
No	210	54944	3.82	301	56020	5.37	1.40 (1.17-1.67)***	1.27 (1.06-1.52)**
Yes	17	2995	5.68	21	4108	5.11	0.91 (0.48-1.73)	0.83 (0.43-1.58)
Chronic obstructive pulmonary disease								
No	124	40896	3.03	171	37603	4.55	1.49 (1.18-1.88)***	1.35 (1.07-1.71)*
Yes	103	17043	6.04	151	22525	6.7	1.11 (0.86-1.42)	1.09 (0.84-1.40)
**Comorbidity amount**								
0	18	11915	1.51	20	6196	3.22	2.06 (1.09-3.91)*	2.15 (1.13-4.09)*
>1	209	46024	4.54	302	53932	5.59	1.23 (1.03-1.47)*	1.19 (1.00-1.42)*

PY, person-years; IR, incidence rate, per 1,000 person-years; HR, hazard ratio; CI, confidence interval.

HR adjusted for gender, age, osteoporosis, diabetes, cirrhosis, cerebrovascular disease, chronic kidney disease, hypertension, hyperlipidemia, coronary artery disease and chronic obstructive pulmonary disease.

*p<0.05

**p<0.01

***p<0.001.

Table 4 presents the incidence rate of AD among different types of AMD. There were no statistically significant differences in the risk of AD between with and without macular degeneration (ICD-9-CM code: 362.50), nonexudative senile macular degeneration (ICD-9-CM code: 362.51), and exudative senile macular degeneration (ICD-9-CM code: 362.52) patients.

**Table 4:** Incidence rates, hazard ratios, and confidence intervals of Alzheimer’s disease among different type of AMD.

Variable	Alzheimer’s disease			Crude HR (95%CI)	Adjusted HR (95%CI)
	Event	PY	IR		
**Type of AMD**					
**ICD-9-CM code: 362.50**					
No	279	65057	4.28	**1(reference)**	**1(reference)**
Yes	270	53010	5.09	1.18 (1.00–1.40)*	1.11 (0.94–1.32)
**ICD-9-CM code: 362.51**					
No	493	109339	4.50	**1(reference)**	**1(reference)**
Yes	56	8728	6.41	1.41 (1.07–1.86)*	1.30 (0.85–1.49)
**ICD-9-CM code: 362.52**					
No	515	112435	4.58	**1(reference)**	**1(reference)**
Yes	34	5632	6.03	1.31 (0.93–1.86)	1.16 (0.82–1.64)

PY, person-years; IR, incidence rate, per 1,000 person-years; HR, hazard ratio; CI, confidence interval.

*p<0.05.

**Fig 1:**
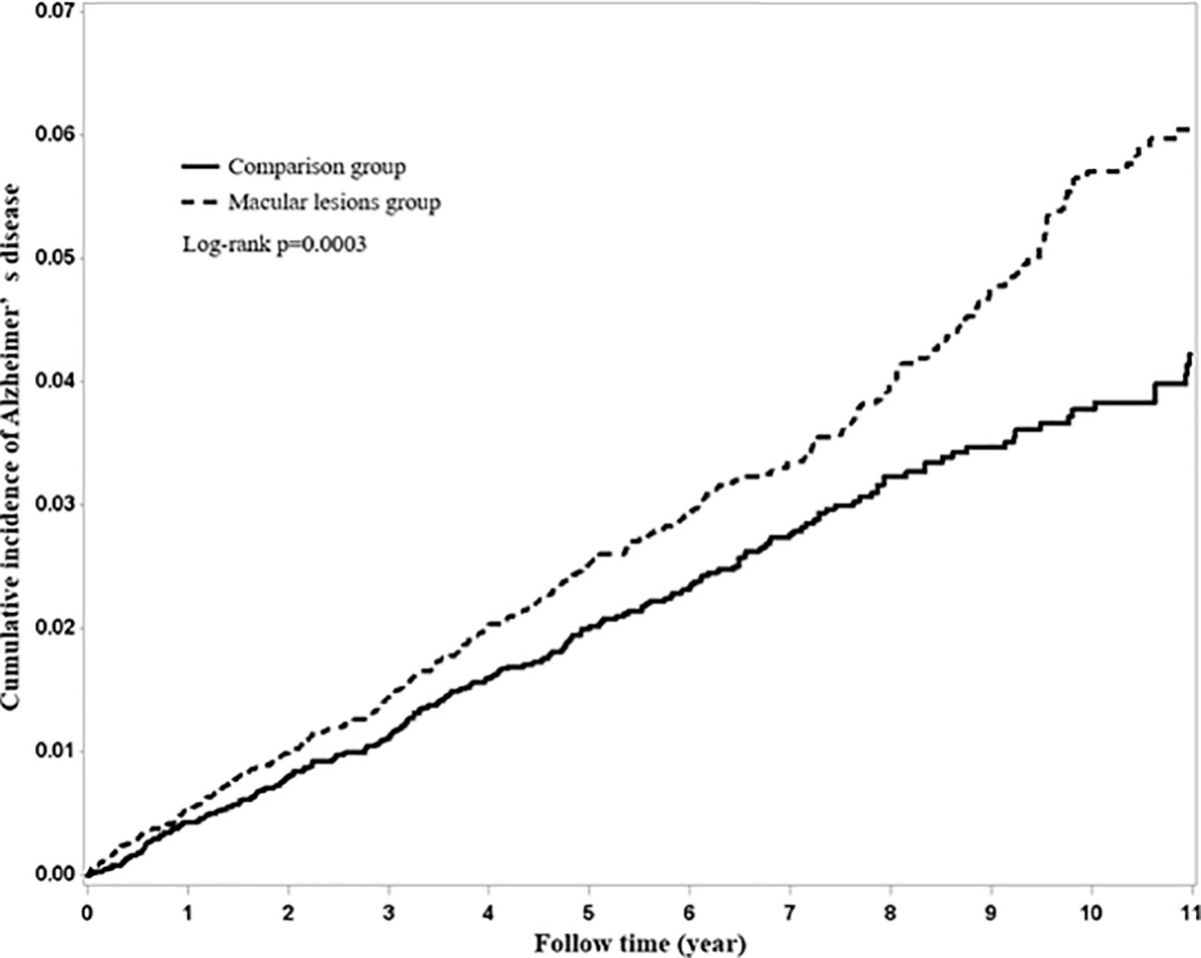
Kaplan-Meier plot of the cumulative incidence of Alzheimer’s disease in patients with and without AMD.

**Fig 2:**
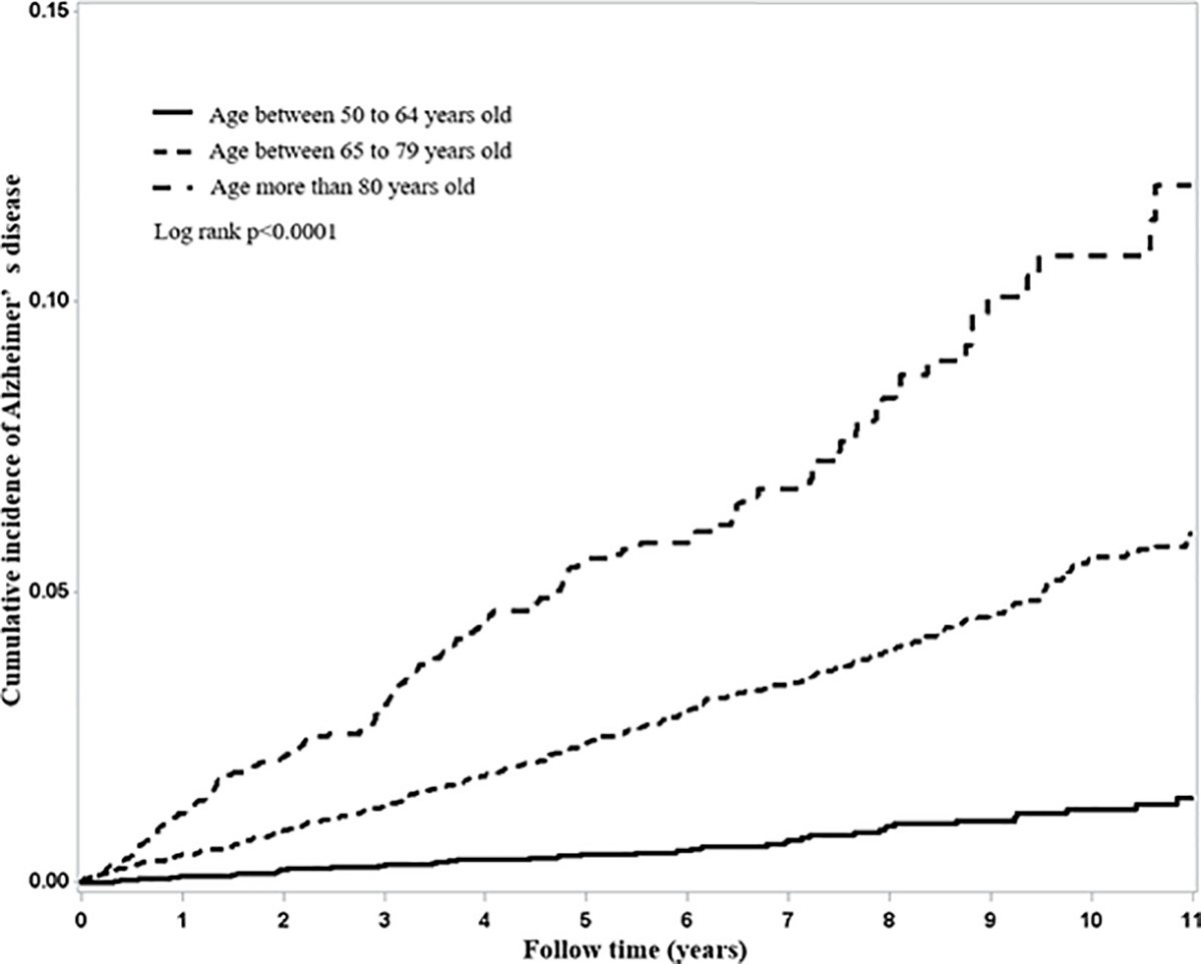
Kaplan-Meier plot of the cumulative incidence of Alzheimer’s disease in each age group.

The Kaplan–Meier plot of the cumulative incidence of Alzheimer’s disease in AMD and comparison cohort is depicted in Fig 1. After 11 years of follow-up, the AMD group had a significantly higher cumulative proportion of AD patients than did the non-AMD group (log-rank test (*p* = 0.003)). Fig 2 presents that there was a considerably different cumulative incidence of among each age group (log-rank *p* <0.0001).

## Discussion

This large-scale, nationwide, retrospective cohort study showed that patients with AMD exhibited a 1.23-fold higher risk of developing AD (aHR = 1.23, 95% CI = 1.04–1.46) than patients without AMD. Old age, cerebrovascular disease, and chronic obstructive pulmonary disease were also risk factors for AD. AMD patients who were female, younger age and with cirrhosis had a significant higher risk of developing AD.

Several publications have documented that AMD is associated with increased risk of AD or cognitive impairment. In 2006, the Blue Mountains Eye Study showed that people with late AMD were associated with lower Mini-Mental State Examination scores than people without AMD [34]. A cross-sectional study reported that AD frequency was significantly increased in the AMD group. The nonexudative AMD subtype was associated with worse cognitive function than the exudative AMD subtype [35]. Similarly, the population-based cohort study in Taiwan by Tsai suggested that patients with AMD had a greater risk of developing AD, although the nonexudative AMD subtype alone showed a conventional level of significance in stratified analysis [36]. However, neither the nonexudative nor the exudative AMD subtype was a significant risk factor for AD in our stratified analysis. The follow-up duration in our study is longer than that of Tsai (2000–2012 vs. 2001–2009), which may contribute to this finding. In a recent paper by Choi, AMD was associated with a higher risk of AD, even after considering lifestyle behaviors, including smoking, alcohol intake, and physical activity [37]. In contrast, the results obtained by Keenan et al. suggested that there is no positive association between AMD and AD. However, in England, most patients with AMD were admitted for receiving intravitreal anti-vascular endothelial growth factor therapy, which is the standard treatment for exudative AMD [30].

Aging is the primary risk factor for both AD and AMD [38]. Oxidative stress is considered to be an imbalance between free radicals and antioxidants, which is the fundamental mechanism contributing to the aging process [39]. Previous studies indicated that oxidative stress may be the common underlying pathological mechanism between AD and AMD [17]. Researchers have found that the levels of lipid peroxidation end products, oxidized proteins, and advanced glycation end products are increased in AD [40, 41]. Likewise, the elevation of oxidative stress was observed in the retina of patients with AMD [42, 43]. In 2011, a study showed that oxidative stress upregulated β-secretase and γ-secretase activities, resulting in increased Aβ production [44]. Common pathogenic pathways could explain the association between AMD and AD.

The strength of this study is the use of NHIRD, a nationwide population-based database, which covers over 99% of medical claims data of citizens in Taiwan, reducing the likelihood of selection and participation bias. However, some limitations should be noted. First, some confounding variables were unavailable in NHIRD. Smoking habit, alcohol consumption, body mass index, physical activity, and family history of AD could not be adjusted in the data analysis. Second, NHIRD lacks imaging findings, neuropsychological assessment results, and laboratory data. Third, AD, AMD, and additional comorbidities cases were identified using ICD-9-CM codes. Same as all claims databases, the accuracy of diagnosis codes is still a concern. However, several studies have validated the positive predictive value of the diagnostic ICD9-CM codes for common diseases, such as ischemic stroke, hypertension, diabetes and all cancers [31–33]. Finally, AD and AMD are not usually diagnosed in the early stages, which could lead to misclassification bias in our study.

In conclusion, our study suggests that patients with AMD have a higher risk of AD than people without AMD. The exact pathological factors are still poorly understood, and further studies need to be conducted to explore the underlying mechanism between AD and AMD.

## Supporting information

S1 Data (DOCX)
