# Supplementary material for: Increased risk of Alzheimer’s disease among patients with age-related macular degeneration: A nationwide population-based study
Source: PLoS One. 2021 May 7;16(5):e0250440. doi: 10.1371/journal.pone.0250440 (PMC8104445; doi:10.1371/journal.pone.0250440)
Supplement: S1 Data — (DOCX) [file pone.0250440.s001.docx]

**Enrollment details (ID)**

| **Variable** | **Label** | **Format** | **Length** | **Description** |
| --- | --- | --- | --- | --- |
| ID | Identification number | Char | 32 | De-identified and anonymous |
| ID_SEX | Sex | Char | 1 | M: Male; F: Female |
| ID_BIRTHDAY | Birthday | Char | 8 | YYYYMMDD |
| ID_IN_DATE | Insurance coverage start date | Char | 8 | YYYYMMDD |
| ID_OUT_DATE | Insurance coverage end date | Char | 8 | YYYYMMDD |

**Outpatient prescription and treatment details (CD)**

| **Variable** | **Label** | **Format** | **Length** | **Description** |
| --- | --- | --- | --- | --- |
| ID | Identification number | Char | 32 | De-identified and anonymous |
| ID_SEX | Sex | Char | 1 | M: Male; F: Female |
| FUNC_DATE | Visit date | Char | 8 | YYYYMMDD |
| ACODE_ICD9_1 | Disease classification code 1 | Char | 15 | ICD-9-CM |
| ACODE_ICD9_2 | Disease classification code 2 | Char | 15 | ICD-9-CM |
| ACODE_ICD9_3 | Disease classification code 3 | Char | 15 | ICD-9-CM |

**Inpatient medical expenses list details (DD)**

| **Variable** | **Label** | **Format** | **Length** | **Description** |
| --- | --- | --- | --- | --- |
| ID | Identification number | Char | 32 | De-identified and anonymous |
| ID_SEX | Sex | Char | 1 | M: Male; F: Female |
| IN_DATE | Admission date | Char | 8 | YYYYMMDD |
| ICD9CM_CODE | Main diagnostic code | Char | 15 | ICD-9-CM |
| ICD9CM_CODE_1 | Second diagnostic code 1 | Char | 15 | ICD-9-CM |
| ICD9CM_CODE_2 | Second diagnostic code 2 | Char | 15 | ICD-9-CM |
| ICD9CM_CODE_3 | Second diagnostic code 3 | Char | 15 | ICD-9-CM |
| ICD9CM_CODE_4 | Second diagnostic code 4 | Char | 15 | ICD-9-CM |
